# The Changing Acceptance of COVID-19 Vaccination in Different Epidemic Phases in China: A Longitudinal Study

**DOI:** 10.3390/vaccines9030191

**Published:** 2021-02-25

**Authors:** Jiahao Wang, Xinran Lu, Xiaozhen Lai, Yun Lyu, Haijun Zhang, Yufei Fenghuang, Rize Jing, Li Li, Wenzhou Yu, Hai Fang

**Affiliations:** 1 School of Public Health, Peking University, Beijing 100083, China; jiahaowang@pku.edu.cn (J.W.); luxr@pku.edu.cn (X.L.); laixiaozhen@pku.edu.cn (X.L.); yun.lyu@bjmu.edu.cn (Y.L.); haijunzhang@pku.edu.cn (H.Z.); yffenghuang@pku.edu.cn (Y.F.); rzjing2015@hsc.pku.edu.cn (R.J.); 2 China Center for Health Development Studies, Peking University, Beijing 100083, China; 3 National Immunization Program, Chinese Center for Disease Control and Prevention, Beijing 102206, China; lili@chinacdc.cn (L.L.); yuwenzhou2012@163.com (W.Y.); 4 Peking University Health Science Center—Chinese Center for Disease Control and Prevention Joint Center for Vaccine Economics, Beijing 100083, China; 5 Key Laboratory of Reproductive Health, National Health Commission of the People’s Republic of China, Beijing 100083, China

**Keywords:** COVID-19, vaccine acceptance, change, China, phase

## Abstract

COVID-19 vaccines have been conditionally used in a few countries, including China since December 2020. The present study aimed to examine whether the acceptance of COVID-19 vaccination changed in different COVID-19 epidemic phases in China. Two consecutive surveys were conducted among Chinese adults in March (*n* = 2058) (severe epidemic phase) and November–December (*n* = 2013) (well-contained phase, right before the COVID-19 vaccine was conditionally approved) 2020, and 791 respondents were longitudinally followed-up. The attitude, acceptance, and preferences for future COVID-19 vaccination were compared between two epidemic phases. Multivariate logistic regression was used to identify influencing factors of acceptance. Among the 791 respondents longitudinally followed, 91.9% in March and 88.6% of them in November–December 2020 would like to get COVID-19 vaccination in China. In March 58.3% wished to get vaccinated immediately, but the proportion declined to 23.0% in November–December 2020, because more respondents wanted to delay vaccination until the vaccine’s safety was confirmed. Similar results were found by comparing all respondents from the two cross-sectional surveys in different epidemic phases. The risk perception, attitude for the importance of vaccination against COVID-19, vaccination history, valuing doctor’s recommendations, vaccination convenience, or vaccine price in decision-making had impacts on respondents’ intention for immediate vaccination. The public acceptance for COVID-19 vaccination in China sustained at a high level in different COVID-19 epidemic phases. However, the intention of immediate vaccination declined substantially due to concerns about the vaccine’s safety. Information about vaccination safety from authoritative sources, doctor’s recommendations, and vaccination convenience were important in addressing vaccine hesitancy and promoting successful herd immunity for the general population in China.

## 1. Introduction

The Coronavirus disease 2019 (COVID-19) pandemic has continued to spread and evolve around the world since its first identification in December 2019 [1,2,3]. As of 25 January 2021, there have been more than 98 million confirmed cases of COVID-19 and two million deaths in 223 countries and regions [2]. In addition to the enormous disease and economic burden posed by COVID-19, the pandemic has brought huge impacts on all aspects of society, such as disrupting economies, social order, and domestic and international communications [2,4,5]. Vaccination against COVID-19 has been regarded as one of the most promising and cost-effective health interventions to prevent and control the pandemic, and vaccines have been developed, tested, and put into use at an unprecedented pace [6,7,8]. So far, 10 leading vaccines have been approved for limited, emergency use or full use in a few countries, such as vaccines developed by Pfizer-BioNTech (in the United States, European Union, and other countries), Oxford-AstraZeneca (in Britain, India, and other countries), Sinopharm, Sinovac, and CanSino (in China) [6,7].

Though vaccines against COVID-19 are available or soon ready for public use, the success of immunization programs, which aim to increase vaccine coverage of the targeted population to achieve herd immunity and a better public health effect, would largely rely on the public attitude and perception of COVID-19 vaccination, especially the willingness to accept vaccination [9,10,11]. What is more, the design and preparation of promotion strategies by governments and other related organizations should be based on the understanding of present public acceptance, so as to ensure the effective and equitable distribution of COVID-19 vaccines for the general population [9,10,11,12,13,14]. So far, some studies concerning the public acceptance of COVID-19 vaccination have been conducted in some countries and regions, and it has been found that the acceptance varied substantially globally [10,11,13,14,15,16,17,18,19,20,21,22,23,24,25]. For example, the acceptance rates could reach about 90% in China, 85% in Brazil, and 80% in South Africa and South Korea, while in countries like Russia and France, the acceptance rates were only about 55% and 60%, respectively [9,10,12,13,15]. Other than the difference between countries, studies in the United States found a declining trend of acceptance of COVID-19 vaccination, as the willingness to vaccinate dropped to about 50% in December 2020 from its peak 74% in April [20,21,22,23,24,25]. This phenomenon is an issue of concern, and the change should also be assessed in other countries around the world, as it may greatly hinder the effect of immunization programs to control the COVID-19 pandemic. In the face of unclear situations and trend of public acceptance, vaccine hesitancy, referred to as the delay in acceptance or refusal of vaccination despite availability of vaccination services, has been frequently pointed out as an underlying obstacle in promoting COVID-19 vaccination programs around the world [26,27,28,29,30]. Previous studies on some other vaccines have shown serious and even ever-lasting impacts of vaccine hesitancy [31,32,33,34]. For example, the misinformation about the association of measles, mumps, and rubella (MMR) vaccine and children autism reported by a discredited study has continually raised the fear among parents for triple MMR vaccination in the United Kingdom and other European countries, resulting in long-lasting low vaccine uptake and waves of local disease outbreaks of, e.g., measles or mumps [31,32,33,34]. Currently, COVID-19 vaccines are developed at an unprecedented pace, while there are increasing antivaccination activities and wide-spreading misinformation about vaccination through various channels. All can contribute to public hesitancy and concern for COVID-19 vaccination, making vaccine hesitancy an all-important problem which needs to be addressed with relevant information from studies in different periods [10,11,13,14,21,26,30].

Despite being hit by COVID-19 the first, China has brought the disease and pandemic under control rapidly and effectively by adopting various measures, such as active case surveillance and management, community screening, quarantine and social distancing, and centralized deployment of medical personnel and resources [35,36,37,38,39]. From mid-February to early March 2020, China reached the severe phase of the COVID-19 pandemic, or the peak of the pandemic. By the end of March, the severe phase came to an end as the number of newly confirmed cases per day dropped to single digits and most of them were imported from overseas [40,41]. Ever since then, China has reached a so-called phase of “regular epidemic prevention and control”, the well-contained phase during which the pandemic was under control, work, study, and life of the general population were resumed, and economic and social order were restored [40]. In addition, as the leading country in the development of COVID-19 vaccines, China approved its first COVID-19 inactivated vaccine developed by a state-owned company Sinopharm on 31 December 2020 for conditional use on the general population, and the vaccines were scheduled to firstly vaccinate high-risk or key groups (e.g., medical professionals) before widening the inoculation to the general public [7,42,43,44]. However, few investigations were available to inform the public perception and acceptance of COVID-19 vaccination in China [9,10,12]. During the severe phase of the pandemic (March 2020), one study by Wang et al. reported an acceptance rate of 91.3% among the Chinese public, with about half (52.2%) who would like to get vaccinated as soon as possible when the vaccine became available [9]. However, these studies did not examine the latest public perception of COVID-19 vaccination and its trend in a different pandemic phase or suggest possible impact factors accordingly due to their cross-sectional design. Other than that, the difference in questionnaire formats hindered the comparability of results [9,10,12].

As the COVID-19 vaccine will soon be available for the general population in China and the immunization program has been considered and prepared since the end of 2020, we conducted a survey in mid-November and December 2020 to assess the latest perception, acceptance and preferences of COVID-19 vaccination in the Chinese public to examine whether the present situation is sufficient for successful promotion of vaccine coverage in China and raise effective measures. In particular, as one of the few countries around the world that have reached the well-contained phase and managed to recover from the pandemic, this study aimed to examine whether the public acceptance and preferences of COVID-19 vaccination have changed in different COVID-19 epidemic phases, as well as its trend. The comparison would help provide empirical evidence for other countries considering tracking public perception of COVID-19 vaccination, and interpreting some key influencing factors in different phases of the COVID-19 pandemic.

## 2. Materials and Methods

### 2.1. Study Design, Population, and Sampling

Two anonymous online surveys were conducted among Chinese adults in March (the severe epidemic phase) and November–December 15 (the well-contained phase) 2020. The study design, target population, sampling method, and source have been reported in a previous study [9]. In general, the two consecutive cross-sectional surveys were conducted using a stratified random sampling method on the biggest online survey platform in China, Wen Juan Xing (Changsha Ranxing Information Technology Co., Ltd., Changsha, Hunan, China). The sample database of the Wen Juan Xing platform consisted of over 2.6 million Chinese members with confirmed personal information and diverse socioeconomic background. The target population are Chinese adults living in mainland China; hence, a random sample procedure stratified by age and location was adopted to match Chinese adults in the Wen Juan Xing sample database. Chinese respondents aged 18 years and above residing in mainland China in the Wen Juan Xing sample database were eligible to participate in the surveys. A total of 2058 respondents were recruited in the first survey in March 2020, and all participants were invited to participate in the follow-up survey in Nov–Dec 2020. Among them, 791 completed the second survey. Additionally, new respondents were recruited in the sample database and 1222 respondents completed the questionnaire, making the total sample size 2013 in the second survey in Nov–Dec 2020. Samples in the two consecutive surveys were merged for analysis, with a longitudinal sample of 791 respondents and a pooled cross-sectional sample of 3280 respondents.

### 2.2. Measures

The design and content of the self-administered questionnaire have been reported in a previous study [9]. Basically, the questionnaire was based on previous studies and frameworks on vaccine acceptance [45,46,47,48]. The contents of the questionnaire included sociodemographic characteristics and vaccination history of the respondents; the impact of the COVID-19 pandemic on respondents’ work/study, income and daily life, perceived risk of being infected personally with COVID-19; acceptance, attitude, preferences for future COVID-19 vaccination, and the importance of identified impact factors on the respondents’ vaccination decision-making, such as vaccine price, vaccination convenience, and doctor’s recommendations. In addition to the previous questionnaire, questions about the preferences of vaccination sites and time, vaccine efficacy, and the duration of hesitancy were added in the present investigation [9]. Most questions were closed-ended and treated as categorical variables, and self-reported questions were assessed on a five-point Likert scale, such as health status, perceived risk of infection, and impact of the COVID-19 pandemic on respondents.

### 2.3. Statistical Analysis

The primary outcome of the consecutive surveys was the acceptance of COVID-19 vaccination [9]. Based on the question “If a COVID-19 vaccine is successfully developed and approved for listing in the future, would you accept vaccination?”, respondents were classified into the vaccine accept group or the refuse group. Those in the accept group were furthered asked the question “Do you want to be vaccinated as soon as possible when the COVID-19 vaccine is available?”, and we classified respondents with the intention of immediate vaccination into the vaccine demand group, and the other who would like to delay into the vaccine delay group. Descriptive statistics were performed and Pearson chi-squared tests were used to examine the change on the attitude, acceptance, and preferences of COVID-19 vaccination in different COVID-19 epidemic phases (the severe epidemic phase and well-contained phase) among respondents of the longitudinal sample (*n* = 791). Additionally, to increase the robustness of the results, the comparison was also conducted between the two cross-sectional samples of March (*n* = 2058) and Nov–Dec (*n* = 2013). To identify the influencing factors of vaccination acceptance, a multivariate logistic regression model was applied between the vaccine demand group and vaccine delay group in the longitudinal sample (balanced panel) (*n* = 791), as well as the pooled cross-sectional samples (unbalanced panel) (*n* = 3280), and Hausman tests were used to check the specification of panel models. The inclusion criteria of variables were reported in a previous study [9]. In general, sociodemographic characteristics, risk perception, impact of COVID-19, vaccination history, attitude towards COVID-19, and impact factors of decision-making were included in regressions, with the vaccine delay group as the reference group. The odds ratio (OR), standard error (SE), and 95% confidence interval (CI) were calculated and reported. All data were analyzed using STATA, version 14.0 (Stata Corp, College Station, TX, USA). Based on the preference of vaccine efficacy, intention of immediate vaccination, and the duration of delaying vaccination among respondents surveyed in Nov–Dec 2020, the possible ranges of public acceptance of COVID-19 vaccination over time under different vaccine efficacy were graphed in the well-contained phase.

## 3. Results

### 3.1. Participant Characteristics

Out of 2058 respondents recruited in the first survey in March 2020, 791 completed the second consecutive survey in Nov–Dec 2020, with a follow-up rate of 38.4%. Table A1 in the Appendix A presents the basic characteristics, risk perception, impact of COVID-19, vaccination history, and impact factors of respondents in the severe epidemic phase (Mar 2020) and well-contained phase (Nov–Dec 2020). Among 791 respondents longitudinally followed-up, more than half of the respondents were 31 to 50 years old (55.4%), and 7.7% were more than 51 years old. Additionally, 53.1% were female, 77.0% were married, 33.0% had a high school or lower level of education and 60.6% had an associate or bachelor’s degree. Nearly half of the respondents (47.1%) had a total annual family income ranging from CNY 100,000 to CNY 150,000 (USD 14,492 to 21,739) in 2019. In Nov–Dec 2020, 85.7% of the respondents were employed, 71.2% were located in Eastern China and 89.5% lived in urban areas. Sixty-nine point eight percent thought that their health status was good or very good. After comparison, the demographic characteristics were similar among respondents in the two cross-sectional surveys of different epidemic phases.

Compared with those in the severe epidemic phase (March 2020), 65.5% of respondents in the well-contained phase (Nov–Dec 2020) in the longitudinal sample stated that there were confirmed or suspected cases in local counties, but 26.0% perceived high or very high risk of COVID-19 infection. The impact of the pandemic on respondents has declined substantially, as 40.2%, 40.2%, and 33.2% thought that the impact of the pandemic on their daily life, work, and income was large or very large now, respectively. In terms of vaccination history, 23.1% have ever refused vaccination with one or more types of vaccines previously. The importance of some factors in respondents’ vaccination decision-making did not differ much. The majority still considered doctor’s recommendation (81.5%) or vaccination convenience (vaccination method, frequency, distance to vaccination sites, etc.) (71.3%) as important factors of their vaccination intention. Over half of the respondents (56.1%) thought that vaccine price was important. Similar trends were observed among respondents in the two cross-sectional surveys of different epidemic phases.

### 3.2. Comparison of Acceptance and Preferences for COVID-19 Vaccination between Two Phases

Table 1 presents the comparison of acceptance, preferences for COVID-19 vaccination between two consecutive surveys in the severe epidemic phase (Mar 2020) and the well-contained phase (Nov–Dec 2020) among respondents in the longitudinal sample as well as the two cross-sectional samples. Among the 791 followed-up respondents, the proportion of general respondents who thought COVID-19 vaccination was an effective way to prevent and control COVID-19 raised to 93.1%, compared with 89.5% in the epidemic phase (*p* = 0.007). However, the intention to accept future COVID-19 vaccination declined from 91.9% to 88.6% in March 2020 with statistical significance (*p* = 0.03). What is worse, when further comparing the intention of immediate vaccination among those in the vaccine accept group in two phases, a substantial decline was observed, as the proportion of respondents who would like to get vaccinated as soon as possible was just 23.0% in Nov–Dec 2020, much lower than 58.3% in Mar (*p* < 0.001). In Nov–Dec 2020, more respondents wanted to delay vaccination until they could confirm the safety of vaccines. In terms of preferences of vaccination, compared with emergency vaccination (13.4%), most respondents would like to get vaccinated with routine immunization schedules in advance of the epidemic (48.5%) or accept both schedules (38.1%). The preference for domestic vaccines increased significantly from 32.3% in March to 48.2% in Nov–Dec 2020, and the preference for imported vaccines remained pretty low (3.4%). Similar reduction of acceptance, intention of immediate vaccination, and trend of preferences were found among general respondents in the two cross-sectional surveys, showing robustness of results.

### 3.3. Influencing Factors of Vaccination Acceptance

Though the consecutive surveys showed a declining trend in the intention to accept future COVID-19 vaccination in the well-contained phase (Nov–Dec 2020) compared with that of the severe epidemic phase (Mar 2020), the majority of respondents (88.6%) still would like to accept vaccination. Therefore, multivariate logistic regression was then performed between the vaccine demand group and vaccine delay group to identify the influencing factors of vaccination acceptance (immediate or delayed acceptance), based on data of the longitudinal sample (balanced panel) (*n* = 791), as well as the pooled cross-sectional samples (unbalanced panel) (*n* = 3280) from the two consecutive surveys. The results of regression models are shown in Table 2, and *p*-values of Hausman tests supported the specification of panel models (the longitudinal sample: 0.62; the pooled cross-sectional samples: 0.48). The regression found that compared with the severe epidemic phase, respondents longitudinally followed-up in the well-contained phase were significantly less likely to accept vaccination as soon as possible (OR: 0.12, 95% CI: 0.08–0.18). Other than that, those perceiving a high or very high risk of infection (OR: 1.59, 95% CI: 1.06–2.40), believing that COVID-19 vaccination was an effective way to prevent and control COVID-19 (OR: 2.07, 95% CI: 1.07–3.99), or valuing doctor’s recommendation as an important factor in vaccination decision-making (OR: 3.13, 95% CI: 1.96–5.01) tended to accept COVID-19 vaccination immediately when the vaccine was available. In contrast, those with confirmed or suspected cases in local counties (OR: 0.69, 95% CI: 0.49–0.96), with history of refusing a certain type of vaccination (OR: 0.57, 95% CI: 0.40–0.82), or with the thought that vaccination convenience (OR: 0.64, 95% CI: 0.46–0.91) or vaccine price (OR: 0.54, 95% CI: 0.40–0.74) was an important factor in vaccination decision-making were less intended to accept immediate vaccination. Similar results were found by regression with the pooled cross-sectional samples, which showed the consistence and robustness of the influencing factors.

### 3.4. Public Preferences for COVID-19 Vaccines and Vaccination and Possible Ranges of Acceptance over Time

Table 3 presents the preferences of vaccination and vaccines among respondents in the vaccine accept group (*n* = 1782), as well the duration of delaying vaccination among those with delayed intention of vaccination (*n* = 1341) surveyed in Nov–Dec 2020, right before the approval of a COVID-19 vaccine in China. Most respondents in the vaccine accept group preferred to get vaccinated at weekends (Saturday or Sunday) (42.7%) or showed no particular preferences. When further asked about their preferred time (day or night), most of them would choose daytime (61.8%) or accept both (36.3%). In terms of preferred vaccination sites, secondary/tertiary hospital (33.5%), local centers for disease control and prevention (29.4), and primary hospital (19.8) were the main choices, while a small portion of respondents also suggested to get vaccinated in temporary vaccination sites (e.g., set in workplaces or schools) (11.7%). Respondents were asked about their willingness to be vaccinated at different levels of vaccine efficacy, and the majority were more willing to be vaccinated at an efficacy of at least 70% (33.2%) or 90% (40.6%). Only a small portion (7.6%) said they were willing to accept vaccination at any level of vaccine efficacy. In the well-contained phase (Nov–Dec 2020), 1341 (66.6%) out of 2013 respondents showed positive but delayed acceptance of vaccination. Most of them (76.9%) said they would wait to see the vaccine’s safety for at least one month (36.9%) or three months (40.0%) before they would receive vaccination.

**Figure 1:**
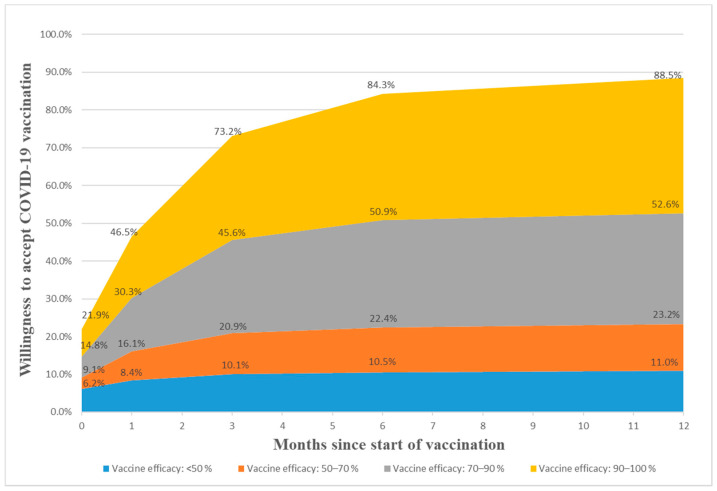
Possible ranges of acceptance of COVID-19 vaccination over time under different vaccine efficacy among respondents (n = 2013) in the well-contained phase (Nov–Dec 2020) in China.

Figure 1 shows the possible ranges of acceptance of COVID-19 vaccination over time under different vaccine efficacies among respondents in the current phase (Nov–Dec 2020). Based on their preferences of vaccine efficacy, decisions on whether to accept immediate vaccination and the duration of delay if they did not want to accept immediate vaccination are reported in this figure. In the best scenario, with vaccine efficacy of 90% or above, the acceptance rate of general respondents reached as high as 14.8–21.9% since the start of vaccination programs and increased to 30.3–46.5% within one month, and 45.6–73.2% in three months. However, the willingness to get COVID-19 vaccination declined to some extent if vaccine efficacy turned out to be lower. For example, if vaccine efficacy was between 70% to 90%, the acceptance rate of general population was about 9.1–14.8% since the start of vaccination programs and increased to 16.1–30.3% within one month, 22.4–50.9% in six months, and below 60% even after one year. In the worst possible scenario, in which vaccine efficacy turned out to be below 50%, the acceptance rate of the general population would reach 10.1% at most after three months, 10.5% at most in six months, and remained low (11.0%) in one year.

## 4. Discussion

To examine the public acceptance of COVID-19 vaccination in China and its change in different COVID-19 epidemic phases, two consecutive surveys were conducted in the severe epidemic phase (Mar 2020) and the well-contained phase (Nov–Dec 2020). Based on the results of respondents in the longitudinal and two cross-sectional samples, it was found that the general acceptance for COVID-19 vaccination in the Chinese population in Nov–Dec 2020, right before the approval of a COVID-19 vaccine, was sustained at a high level (88.5%), though with a reduction compared with the severe epidemic phase (March 2020) (91.9%) [9]. However, the intention of accepting immediate vaccination after the vaccine is available has declined substantially, from 52.2% in Mar 2020 to 24.7% in Nov–Dec 2020 due to concerns about vaccine safety [9]. In addition, the ranges of acceptance over time in the general population varied widely with different vaccine efficacy, and respondents’ preferences for COVID-19 vaccines and vaccination were observed. Risk perception, positive attitude on the importance of vaccination against COVID-19, vaccination history, valuing doctor’s recommendations, vaccination convenience, or vaccine price in decision-making had an effect on the intention for immediate vaccination among respondents.

So far, three studies have investigated the public acceptance of COVID-19 in China, one (the first round of our consecutive surveys) in the severe epidemic phase (March), and the other two in May and June [9,10,12]. The highest acceptance rate was observed at the peak of the pandemic (91.3%), and the other two were 83.3% and 90%, respectively [9,10,12]. The latest results of acceptance (88.5%) in Nov–Dec 2020 suggested declined acceptance in the well-contained phase, but the reduction was not large. In contrast, existing studies in some western countries, such as Italy and the United States, showed a substantial reduction of public acceptance rates as the pandemic progressed over different phases [14,18,20,21,22,23,24,25]. For example, the willingness to vaccinate in the United States has dropped by 24% from April (74%) to December (50%) 2020 [21,23,24,25]. Additionally, the acceptance of vaccination in China was found to remain high compared with other countries around the world, even in the well-contained phase, where the pandemic was effectively controlled [10,11,13,14,15,16,17,18,19,25]. For example, studies reported that public acceptance of COVID-19 vaccination ranged from 62% to 80% in some European countries, among which Denmark and the United Kingdom had the highest acceptance (80%), while France (58.9–62%) and Italy (59%) had the lowest [13,14,15]. In Asian regions, the acceptance was relatively higher, as shown in South Korea (79.8%), Indonesia (67.0–93.3%), and Malaysia (94.3%) [16,19]. To interpret the difference of acceptance across countries, many factors should be considered, including social, cultural, and political contexts, the control and impact of the pandemic, public perception of infection risk and importance of vaccination, as well as public health literacy and trust in governments [9,10,14,23,24,25,26,30]. Based on our findings, the majority of the public (93.1%) in China had a positive attitude towards vaccination and considered it as an effective way to prevent and control COVID-19. Additionally, respondents’ daily life, work and study were still hindered by the pandemic to a significant extent (37.4–41.1%), and all would contribute to high willingness to accept COVID-19 vaccination.

However, the high level of acceptance and positive attitude in the current phase would not guarantee successful a vaccination campaign in the general population in China if we considered vaccine hesitancy [27,28,29]. When asked about whether they would get vaccinated as soon as possible, less than 25% of respondents in the phase Nov–Dec 2020 had the intention of immediate vaccination in both the longitudinal and cross-sectional samples, with a reduction as high as 50% compared with the results in March 2020. Vaccine hesitancy of COVID-19 vaccination has become prevailing around the world [10,11,13,14,17]. For example, 31.6% of respondents in the United States and 18.9% in seven European countries were unsure whether to be vaccinated [13,21]. Concerns about vaccine safety or side effects were reported as the predominant reason for the hesitancy, and previous studies on people’s vaccination acceptance against severe newly emerging infectious diseases (e.g., H1N1 influenza) also stressed that uncertainties about new vaccines, especially the safety, would lower vaccine confidence and therefore the acceptance [11,13,21,49,50]. In our study, during the time when the COVID-19 vaccine and vaccination programs are soon becoming available for the public, the concern of vaccine safety has increased and drives the majority (75.3%) of respondents to delay their vaccination. It would take most (76.9%) of the delayers at least one to three months to make decisions on inoculation, which hindered the transformation from vaccination intention to real uptake, and reduced the effect of immunization programs on controlling the pandemic at the quickest pace. Therefore, public concerns for vaccine safety should also be considered as a priority issue in future vaccination campaigns. In addition to vaccine safety, other vaccine attributes were also reported as important predictors of vaccine acceptance and uptake [11,16,19,20,22]. One study in Indonesia reported that the acceptance of the public would decline from 93.3% with 95% vaccine effectiveness to 67% with 50% effectiveness [19]. We also found strong preference for higher vaccine efficacy in the Chinese population, as the acceptance of COVID-19 vaccination and its trend over time declined substantially with lower vaccine efficacy. If the efficacy of vaccines was assumed to be 50%, the lowest standard for COVID-19 vaccines to be approved for listing by WHO, China, the United States, and other countries, the acceptance rate of the general population would be as low as 10.5% at most in six months and remain below 11.0% in one year. It would be a big threat for a successful national vaccination campaign with the aim of increasing the coverage rate and reaching a herd immunity effect [51,52,53,54]. This finding is consistent with one discrete choice experiment (DCE) survey, which pointed out that the Chinese public strongly preferred high effectiveness of the COVID-19 vaccine, followed by long protective duration and very few adverse events [55]. Many factors also contributed to people’s vaccination decision-making. Similar to other studies, we found that risk perception of the disease, history and attitude of vaccination, doctor recommendation, as well as vaccine price and vaccination convenience are influencing factors on vaccine acceptance [9,11,19,20,22].

On December 30, 2020, China gave a conditional approval for public use of an inactivated COVID-19 vaccine developed by a state-owned company, Sinopharm, which reported a 79% efficacy rate by interim data in phase 3 trials [42]. China has initiated vaccination programs firstly among key groups and those at higher risk of infection aged 18–59 (e.g., workers in the cold-chain logistics sector, customs inspectors, health professionals, community workers) [43]. As further approval and supply of the vaccines are prepared in the future, mass vaccination will then cover other high-risk groups (e.g., the elderly and those with underlying diseases) and lastly, the general population. COVID-19 vaccination would be provided for free to all Chinese citizens [42,43,56]. However, based on our findings in Nov–Dec 2020, right before the approval of the vaccine, the projected coverage rate was unsatisfying if no further interventions were implemented. With 79% efficacy, the acceptance rate would reach 22.4–50.9% in six months and be below 52.6% in one year since the start of vaccination, which is still less than the estimated rate for herd immunity [54]. Hence, to control COVID-19 and restore social activities in an effective and rapid manner, proper vaccination strategies and immunization programs should be designed to increase the coverage, especially among those with vaccine hesitancy. As public concern about vaccine safety appeared to be an obvious obstacle for the rapid progress of vaccine uptake, comprehensive planning and measures are needed to address this issue throughout vaccination programs [57,58]. A better communicative environment with smooth and effective exchange channels should be constructed among the public, health professionals and authorities, governments, and other sections [57,59,60,61]. It is suggested to keep track of and listen to the change of public concerns and sentiments via traditional and social media and detect potential misinformation or conspiracy theories which would hinder the buildup of positive perceptions about vaccination. Trusted authorities like medical professionals, governments, or other sources should actively organize health education and communication to combat disinformation and misinformation and spread authoritative information in a transparent way, especially information about vaccine effectiveness and adverse events [9,10,14,57,62,63]. In particular, it would be of great help to encourage medical professionals, community leaders, and friends or relatives around to share their personal experiences about COVID-19 vaccination to build vaccine confidence and trust [57,62,63]. In addition, a national policy targeting adverse events after COVID-19 vaccination (i.e., disabilities, deaths) is also needed to compensate for people’s losses. Previous lessons and experience in preventing and controlling severe infectious diseases (e.g., H1N1 influenza, the current COVID-19) have shown that after the vaccines were available to the public, the continuing post-marketing surveillance and timely disclosure of related information were of great importance for public confidence, vaccination decision-making, and the success of vaccination programs [58,64,65]. It has also been discussed or piloted to design some regulations or laws to connect vaccination with other affairs such as school attendance [61,66]. In addition to addressing vaccine hesitancy, the promotion of vaccine administration system capability and design of immunization schedules should consider the public preferences of the vaccine and vaccination, such as time, place, and vaccine type. Currently, guidelines for COVID-19 vaccination program of key groups in China are under discussion in terms of the setting of vaccination sites, training of medical personals, monitoring of adverse reactions, and emergency treatment, and the guidelines will serve as a base for a future vaccination program for the public [38]. As shown in our study, the public is more likely to get vaccinated with routine immunization schedules in advance of the epidemic (50.7%), and the preference for imported vaccines remained pretty low (3.3%) [9]. Additionally, based on respondents’ preferences, the resources (e.g., equipment, medical staff, and supply of vaccines) of vaccination should be well-prepared or deployed in hospitals and local centers for disease control and prevention in weekends and daytime. In addition, public infrastructures such as convention centers and sports stadiums could be used to vaccinate a huge amount of people without crowding, and China had lots of experience in making full use of these facilities. These measures would help encourage the public to receive vaccination by enhancing convenience and accessibility.

This study assessed the change of acceptance and preferences of COVID-19 vaccination among in Chinese population in different COVID-19 epidemic phases, including the severe epidemic phase (Mar 2020) and the well-contained phase (Nov–Dec 2020). The present study designed two consecutive surveys to collect longitudinal sample data, which was conducive to the comparison of acceptance in different phases and the identification of related impact factors. The design and results of our study could serve as a reference for other countries in analyzing the public perception and hesitancy of COVID-19 vaccination, interpreting some key influencing factors in different phases of the COVID-19 pandemic, and suggesting effective and proper interventions for future vaccination campaigns. Our study also has several limitations. Firstly, the use of an online survey may limit the representativeness of the results. The large sample size and random stratified sampling method was adopted to try to address this limitation. Secondly, the study was conducted before the approval of a COVID-19 vaccine in China, and little information about the vaccine, such as efficacy, safety, or protective duration, was available. The assessed acceptance may differ from real practices and be changed by the influence of vaccine attributes, the evolution of the pandemic, as well as other factors. Further studies and investigations are suggested in monitoring the public perception, acceptance, and uptake of COVID-19 vaccination after the launch of a national vaccination program, taking measures to promote inoculation capability and vaccine supply, and assessing the effects of vaccination programs in terms of access, distribution, coverage, and equity.

## 5. Conclusions

Based on results from two consecutive surveys, the present study found that public acceptance for COVID-19 vaccination in China was sustained at a high level in both the severe pandemic phase in March 2020 and the well-contained phase in Nov–Dec 2020. However, due to concerns about vaccine safety, a substantial decline in the intention of immediate vaccination was observed. Effective measures should be designed and taken in the coming immunization program for the general population. Public information about vaccination safety from authoritative sources, doctor’s recommendations, and vaccination convenience are important in addressing vaccine hesitancy and promoting successful herd immunity for the general population in China.

## Figures and Tables

**Table 1:** Comparison of acceptance and preferences for COVID-19 vaccination between two consecutive surveys in the severe epidemic phase (Mar 2020) and the well-contained phase (Nov–Dec 2020).

Items	Longitudinal Sample			Cross-Sectional Samples		
	Mar 2020 (Severe epidemic Phase)	Nov–Dec 2020 (Well-contained Phase)		Mar 2020 (Severe epidemic Phase)	Nov–Dec 2020 (Well-contained Phase)	
	N (%)	N (%)	*p*-value	N (%)	N (%)	*p*-value
**Overall respondents**	791 (100)	791 (100)		2058 (100)	2013 (100)	
COVID-19 vaccination is an effective way to prevent and control COVID-19						
Yes	718 (90.8)	746 (94.3)		1842 (89.5)	1874 (93.1)	
No	73 (9.2)	45 (5.7)	0.007	216 (10.5)	139 (6.9)	<0.001
Accept vaccination if the COVID-19 vaccine is successfully developed and approved for listing in the future						
Yes	727 (91.9)	701 (88.6)		1879 (91.3)	1782 (88.5)	
No	64 (8.1)	90 (11.4)	0.03	179 (8.7)	231 (11.5)	0.003
**Vaccine accept group**	727 (100)	701 (100)		1879 (100)	1782 (100)	
Want to receive vaccination as soon as possible when the vaccine is available						
Yes, as soon as possible	424 (58.3)	161 (23.0)		980 (52.2)	441 (24.7)	
No, delay vaccination until I confirmed the vaccine safety	303 (41.7)	540 (77.0)	<0.001	899 (47.8)	1341 (75.3)	<0.001
Prefer which kind of immunization schedules of the COVID-19 vaccination						
Routine immunization	333 (45.8)	340 (48.5)		928 (49.4)	904 (50.7)	
Emergency vaccination	79 (10.9)	94 (13.4)		169 (9.0)	223 (12.5)	
Both are acceptable	315 (43.3)	267 (38.1)	0.09	782 (41.6)	655 (36.8)	<0.001
Prefer which type of COVID-19 vaccines						
Domestic vaccine	235 (32.3)	338 (48.2)		611 (32.5)	975 (54.7)	
Imported vaccine	30 (4.1)	24 (3.4)		62 (3.3)	59 (3.3)	
Both are acceptable	462 (63.6)	339 (48.4)	<0.001	1206 (64.2)	748 (42.0)	<0.001

**Table 2:** Influencing factors of vaccination acceptance (immediate or delayed acceptance) between the vaccine demand group and vaccine delay group.

Characteristics	Longitudinal Sample				Pooled Cross-Sectional Sample					
	OR	SE	*p*-Value	95% CI	OR	SE	*p*-Value	95% CI
Phase								
Severe epidemic phase	Ref				Ref			
Well-contained phase	0.12	0.02	<0.001	(0.08, 0.18)	0.19	0.03	<0.001	(0.14, 0.25)
Age group								
18~25	Ref				Ref			
26~30	1.19	0.36	0.58	(0.65, 2.15)	1.13	0.20	0.50	(0.80, 1.59)
31~40	1.35	0.41	0.32	(0.74, 2.47)	1.07	0.20	0.71	(0.75, 1.53)
41~50	1.39	0.44	0.31	(0.74, 2.59)	1.06	0.20	0.77	(0.73, 1.53)
>51	1.79	0.79	0.19	(0.75, 4.27)	1.14	0.28	0.59	(0.70, 1.86)
Gender								
Female	Ref				Ref			
Male	1.15	0.18	0.37	(0.85, 1.55)	1.29	0.12	0.01	(1.07, 1.54)
Highest level of education								
Middle school and below	Ref				Ref			
High school	1.71	0.79	0.25	(0.69, 4.25)	0.92	0.19	0.67	(0.61, 1.38)
Associate or Bachelor	1.45	0.69	0.43	(0.57, 3.67)	0.85	0.18	0.45	(0.56, 1.29)
Master and above	1.08	0.61	0.89	(0.36, 3.24)	0.79	0.22	0.39	(0.45, 1.36)
Marriage status								
Others (Single, Divorced or Widowed)	Ref				Ref			
Married	1.48	0.33	0.08	(0.95, 2.30)	1.78	0.25	<0.001	(1.35, 2.35)
Location								
Central	Ref				Ref			
East	0.65	0.13	0.03	(0.44, 0.96)	0.85	0.09	0.13	(0.68, 1.05)
West	0.64	0.17	0.10	(0.37, 1.09)	0.94	0.14	0.67	(0.71, 1.25)
Region								
Rural	Ref				Ref			
Urban	0.53	0.12	0.01	(0.34, 0.84)	0.85	0.10	0.18	(0.67, 1.08)
Employment status								
Unemployed	Ref				Ref			
Employed	0.99	0.76	0.99	(0.22, 4.44)	0.74	0.31	0.48	(0.33, 1.69)
Health status								
Fair or below (fair, poor, very poor)	Ref				Ref			
Good and above (good, very good)	1.18	0.21	0.33	(0.84, 1.66)	1.13	0.12	0.23	(0.92, 1.38)
Total family income in 2019								
≤CNY 50,000	Ref				Ref			
CNY 50,000–100,000	0.90	0.31	0.76	(0.46, 1.75)	0.66	0.11	0.01	(0.48, 0.91)
CNY 100,000–150,000	0.96	0.33	0.90	(0.49, 1.87)	0.61	0.10	<0.001	(0.44, 0.86)
CNY 150,000–200,000	0.69	0.25	0.30	(0.34, 1.39)	0.66	0.12	0.02	(0.46, 0.95)
CNY 200,000–300,000	1.25	0.48	0.55	(0.60, 2.64)	0.76	0.15	0.18	(0.51, 1.13)
≥CNY 300,000	1.61	0.67	0.25	(0.71, 3.64)	1.03	0.24	0.90	(0.66, 1.62)
Refused vaccination of a certain type of vaccine in the past								
No	Ref				Ref			
Yes	0.57	0.10	<0.001	(0.40, 0.82)	0.78	0.09	0.03	(0.63, 0.97)
There are confirmed or suspected cases in the county								
No or not clear	Ref				Ref			
Yes	0.69	0.12	0.03	(0.49, 0.96)	0.76	0.08	0.01	(0.62, 0.92)
Perceived risk of infection								
Fair	Ref				Ref			
High or very high	1.59	0.33	0.03	(1.06, 2.40)	1.83	0.25	<0.001	(1.41, 2.38)
Low or very low	0.87	0.15	0.43	(0.63, 1.22)	1.06	0.11	0.58	(0.86, 1.30)
Pandemic impact on daily life								
Fair	Ref				Ref			
Large or very large	0.93	0.17	0.70	(0.65, 1.33)	1.05	0.12	0.64	(0.85, 1.31)
Small or very small	0.95	0.24	0.85	(0.58, 1.56)	0.89	0.14	0.47	(0.66, 1.21)
Pandemic impact on work								
Fair	Ref				Ref			
Large or very large	0.94	0.19	0.77	(0.64, 1.39)	1.12	0.14	0.35	(0.88, 1.43)
Small or very small	0.77	0.20	0.33	(0.46, 1.30)	0.99	0.17	0.96	(0.71, 1.38)
Pandemic impact on income								
Fair	Ref				Ref			
Large or very large	1.05	0.19	0.79	(0.73, 1.51)	1.00	0.12	0.98	(0.79, 1.26)
Small or very small	0.80	0.18	0.31	(0.51, 1.24)	0.79	0.11	0.10	(0.59, 1.05)
COVID-19 vaccination is an effective way to prevent and control COVID-19								
No	Ref				Ref			
Yes	2.07	0.69	0.03	(1.07, 3.99)	1.89	0.36	<0.001	(1.30, 2.74)
Doctor’s recommendation is an important factor in vaccination decision-making								
No	Ref				Ref			
Yes	3.13	0.75	<0.001	(1.96, 5.01)	2.72	0.39	<0.001	(2.06, 3.59)
Vaccination convenience is an important factor in vaccination decision-making								
No	Ref				Ref			
Yes	0.64	0.11	0.01	(0.46, 0.91)	0.59	0.07	<0.001	(0.47, 0.74)
Vaccine price is an important factor in vaccination decision-making								
No	Ref				Ref			
Yes	0.54	0.09	<0.001	(0.40, 0.74)	0.53	0.05	<0.001	(0.44, 0.65)

**Table 3:** Preferences for COVID-19 vaccination among respondents in the well-contained phase (Nov–Dec 2020) in China.

Items	N (%)
**Vaccine accept group**	1782 (100)
Prefer when to get vaccinated (weekday/weekend)	
Weekday (Monday to Friday)	150(8.4)
Weekend (Saturday or Sunday)	761(42.7)
Both are acceptable	871(48.9)
Prefer when to get vaccinated (day/night time)	
Day time	1102 (61.8)
Night time	34 (1.9)
Both are acceptable	646 (36.3)
Prefer where to get vaccinated	
Secondary/ Tertiary hospital	597 (33.5)
Primary hospital	353 (19.8)
Local centers for disease control and prevention	524 (29.4)
Temporary vaccination sites (e.g., set in workplace or school)	208 (11.7)
Residents’ committee/ Villagers’ committees	94 (5.3)
Private hospital	6 (0.3)
Willing to accept vaccination at what level of vaccine efficacy	
Willing to accept vaccination at any level	135 (7.6)
At least 30%	87 (4.9)
At least 50%	246 (13.8)
At least 70%	591 (33.2)
At least 90%	723 (40.6)
**Vaccine delay group**	1341 (100)
The duration of delaying vaccination to see the vaccine safety	
At least 1 month	495 (36.9)
At least 3 months	537 (40.0)
At least 6 months	224 (16.7)
At least 1 year	85 (6.3)

## Data Availability

The data presented in this study are available on request from the corresponding author. The data are not publicly available due to privacy.

## Appendix A

**Table A1:** Basic characteristics, risk perception, impact of COVID-19, vaccination history, and impact factors among respondents in two consecutive surveys in the severe epidemic phase (Mar 2020) and the well-contained phase (Nov–Dec 2020).

Items	Longitudinal Sample (*n* = 791)		Cross-Sectional Sample		
	Mar 2020 (Severe epidemic Phase)	Nov–Dec 2020 (Well-contained Phase)	Mar 2020 (Severe epidemic Phase) (*n* = 2058)		Nov–Dec 2020 (Well-contained Phase) (*n* = 2013)
	N (%)	N (%)	N (%)		N (%)
Age group					
18–25	119 (15.0)	119 (15.0)	475 (23.1)		332 (16.5)
26–30	173 (21.9)	173 (21.9)	400 (19.4)		434 (21.6)
31–40	260 (32.9)	260 (32.9)	523 (25.4)		717 (35.6)
41–50	178 (22.5)	178 (22.5)	510 (24.8)		360 (17.9)
51 and above	61 (7.7)	61 (7.7)	150 (7.3)		170 (8.4)
Gender					
Female	420 (53.1)	420 (53.1)	1115 (54.2)		987 (49.0)
Male	371 (46.9)	371 (46.9)	943 (45.8)		1026 (51.0)
Highest level of education					
Middle school and below	27 (3.4)	27 (3.4)	123 (6.0)		111 (5.5)
High school	234 (29.6)	234 (29.6)	663 (32.2)		585 (29.1)
Associate or bachelor	479 (60.6)	479 (60.6)	1140 (55.4)		1214 (60.3)
Master and above	51 (6.5)	51 (6.5)	132 (6.4)	103 (5.1)	
Marriage status					
Married	592 (74.8)	609 (77.0)	1385 (67.3)		1456 (72.3)
Others (single, divorced or widowed)	199 (25.2)	182 (23.0)	673 (32.7)		557 (27.7)
Location					
Central	154 (19.5)	136 (17.2)	531 (25.8)		409 (20.3)
East	536 (67.8)	563 (71.2)	1195 (58.1)		1311 (65.1)
West	101 (12.8)	92 (11.6)	332 (16.1)		293 (14.6)
Region					
Rural	113 (14.3)	83 (10.5)	420 (20.4)		333 (16.5)
Urban	678 (85.7)	708 (89.5)	1638 (79.6)		1680 (83.5)
Employment status					
Employed	695 (87.8)	678 (85.7)	1651 (80.2)		1714 (85.1)
Unemployed	96 (12.2)	113 (14.3)	407 (19.8)		299 (14.9)
Health status					
Good and above (good, very good)	594 (75.1)	552 (69.8)	1527 (74.2)		1366 (67.9)
Fair or below (fair, poor, very poor)	197 (24.9)	239 (30.2)	531 (25.8)		647 (32.1)
Total annual family income in 2019					
≤CNY 50,000 (USD 7246)	56 (7.1)	56 (7.1)	277 (13.4)		207 (10.3)
CNY 50,000–100,000 (USD 7246–14,492)	139 (17.6)	139 (17.6)	548 (26.6)		490 (24.3)
CNY 100,000–150,000 (USD 14,492–21,739)	191 (24.2)	191 (24.2)	506 (24.6)		489 (24.3)
CNY 150,000–200,000 (USD 21,739–28,986)	181 (22.9)	181 (22.9)	352 (17.1)		395 (19.6)
CNY 200,000–300,000 (USD 28,986–43,478)	139 (17.6)	139 (17.6)	239 (11.7)		284 (14.1)
≥CNY 300,000 (USD 43,478)	85 (10.7)	85 (10.7)	136 (6.6)		148 (7.4)
There are confirmed or suspected cases in the county					
Yes	618 (78.1)	518 (65.5)	1538 (74.7)		1282 (63.7)
No or not clear	173 (21.9)	273 (34.5)	520 (25.3)		731 (36.3)
Perceived risk of infection					
High or very high	104 (13.2)	206 (26.0)	251 (12.2)		498 (24.8)
Fair	238 (30.1)	256 (32.4)	575 (27.9)		589 (29.3)
Low or very low	449 (56.8)	329 (41.6)	1232 (59.9)		926 (46.0)
Pandemic impact on daily life					
Large or very large	550 (69.5)	318 (40.2)	1368 (66.5)		844 (41.9)
Fair	173 (21.9)	269 (34.0)	497 (24.1)		651 (32.3)
Small or very small	68 (8.6)	204 (25.8)	193 (9.4)		518 (25.7)
Pandemic impact on work					
Large or very large	533 (67.4)	318 (40.2)	1326 (64.4)		828 (41.1)
Fair	145 (18.3)	244 (30.8)	402 (19.5)		583 (29.0)
Small or very small	72 (9.1)	174 (22.0)	191 (9.3)		445 (22.1)
Missing	41 (5.2)	55 (6.9)	139 (6.8)		157 (7.8)
Pandemic impact on income					
Large or very large	388 (49.1)	263 (33.2)	905 (44.0)		753 (37.4)
Fair	203 (25.6)	252 (31.9)	467 (22.7)		567 (28.2)
Small or very small	129 (16.3)	191 (24.1)	325 (15.8)		458 (22.8)
Missing	71 (9.0)	85 (10.8)	361 (17.5)		235 (11.7)
Refused vaccination of a certain type of vaccine in the past					
Yes	183 (23.1)	183 (23.1)	459 (22.3)		437 (21.7)
No	608 (76.9)	608 (76.9)	1599 (77.7)		1576 (78.3)
Doctor’s recommendation is an important factor in vaccination decision-making					
Yes	659 (83.3)	645 (81.5)	1659 (80.6)		1625 (80.7)
No	132 (16.7)	146 (18.5)	399 (19.4)		388 (19.3)
Vaccination convenience (vaccination method, frequency, distance to vaccination sites, etc.) is an important factor in vaccination decision-making					
Yes	577 (72.9)	564 (71.3)	1558 (75.7)		1432(71.1)
No	214 (27.1)	227 (28.7)	500 (23.3)		581 (28.9)
Vaccine price is an important factor in vaccination decision-making					
Yes	452 (57.1)	444 (56.1)	1233 (59.9)		1197 (59.5)
No	339 (42.9)	347 (43.9)	825 (40.1)		816 (40.5)
